# The Spatiotemporal Data Fusion (STDF) Approach: IoT-Based Data Fusion Using Big Data Analytics

**DOI:** 10.3390/s21217035

**Published:** 2021-10-23

**Authors:** Dina Fawzy, Sherin Moussa, Nagwa Badr

**Affiliations:** Information Systems Department, Faculty of Computer and Information Sciences, Ain Shams University, Cairo 11566, Egypt; dina.fawzy@cis.asu.edu.eg (D.F.); nagwabadr@cis.asu.edu.eg (N.B.)

**Keywords:** Internet of Things, big data analytics, data fusion, real-time processing, data reduction, data aggregation, cluster sampling

## Abstract

Enormous heterogeneous sensory data are generated in the Internet of Things (IoT) for various applications. These big data are characterized by additional features related to IoT, including trustworthiness, timing and spatial features. This reveals more perspectives to consider while processing, posing vast challenges to traditional data fusion methods at different fusion levels for collection and analysis. In this paper, an IoT-based spatiotemporal data fusion (STDF) approach for low-level data in–data out fusion is proposed for real-time spatial IoT source aggregation. It grants optimum performance through leveraging traditional data fusion methods based on big data analytics while exclusively maintaining the data expiry, trustworthiness and spatial and temporal IoT data perspectives, in addition to the volume and velocity. It applies cluster sampling for data reduction upon data acquisition from all IoT sources. For each source, it utilizes a combination of k-means clustering for spatial analysis and Tiny AGgregation (TAG) for temporal aggregation to maintain spatiotemporal data fusion at the processing server. STDF is validated via a public IoT data stream simulator. The experiments examine diverse IoT processing challenges in different datasets, reducing the data size by 95% and decreasing the processing time by 80%, with an accuracy level up to 90% for the largest used dataset.

## 1. Introduction

The Internet of Things (IoT) is an emerging technology that connects various objects in the physical world in order to communicate and exchange data [1,2]. It plays a vital role in different practical systems for decision support and control by providing intelligent services and applications as a major source of big data [3,4]. Examples include the healthcare, environment, transportation, human-based and energy fields, where many sensors and devices are deployed to sense and perceive various data [5]. 

However, the multiple sources, heterogeneity and large volumes of unreliable data collected at unprecedented speeds make receiving all data impossible, which obviously consumes a high network bandwidth and device power [6]. Hence, data fusion has become a significant approach to extract and integrate critical data from widely sensed multimodal sources and types in a uniform manner [7]. Therefore, data fusion can reduce the size and dimensions of data, optimize data quality and extract useful information from them [8]. It helps in eliminating data imperfections and handles the sensed data heterogeneity from different sources [9]. Although IoT has provided huge benefits, it has posed many challenges to typical data fusion approaches, due to the new data perspectives introduced by IoT data, such as data expiry, semantics, trustworthiness, accuracy and spatiotemporality [10].

Furthermore, the processing complexity of IoT data has allowed data fusion at three levels in IoT-based systems: (1) low-level data fusion, where the raw data generated from physical objects are fed directly into the fusion process to provide better information; (2) middle-level data fusion, where different features from heterogeneous raw data are fused to find relevant features extracted from diverse data fusion methods; and (3) high-level data fusion, which provides decisions fused from input decisions to obtain the most optimum one [11]. In practice, any combination of these three levels can be employed: data in–data out, data in–feature out, feature in–feature out, feature in–decision out, decision in–decision out. 

The scope of this study focuses on low-level data fusion: data in–data out fusion directly from IoT sources. This provides ready-fused data streams, which can be considered for further intended business purposes and domain-specific applications to obtain a domain-specific data out, feature out or decision out. Thus, the main contributions of this paper can be summarized as follows:

1. We propose the spatiotemporal data fusion (STDF) approach for IoT data;
2. To the best of our knowledge, STDF is the first data in–data out (DAI–DAO) data fusion approach for IoT data that is independent of any IoT domain;
3. To the best of our knowledge, STDF is the first data fusion approach for IoT data that preserves the spatial and temporal characteristics of IoT data during fusion, considering all timing characteristics of IoT data;
4. STDF uniquely investigates predefined and trusted IoT data sources to ensure private IoT data fusion;
5. STDF grants optimum performance for big IoT data fusion, which effectively supports scalable real-time processing of such vastly generated IoT data;
6. STDF ensures accurate IoT data fusion, considering both big data and IoT-related data characteristics;
7. A detailed experimental analysis is conducted to evaluate the proposed STDF approach using a publicly available IoT data stream simulator for validity.

The remaining sections of this paper are structured as follows. Section 2 evaluates the related works of data fusion approaches considered for different IoT systems. Section 3 discusses the problem definition and the main contributions of this study. Section 4 presents the proposed approach, with a detailed description of its layers and modules. Section 5 discusses the experimental methodology and resultant outcomes to evaluate the proposed approach, whereas Section 6 presents a detailed discussion of the experimental results. Finally, Section 7 concludes our study and highlights future work.

## 2. Related Works

**Figure 1:**
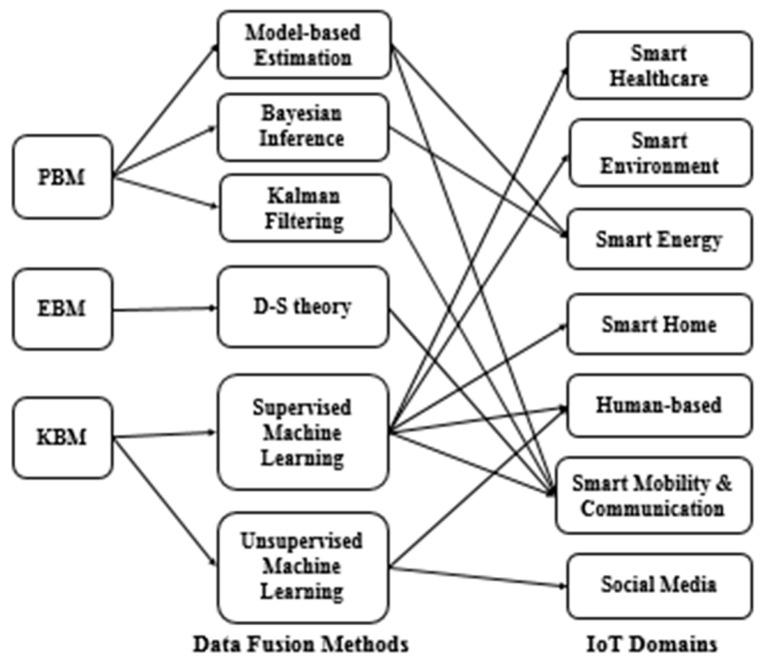
Data fusion methods considered for different IoT domains.

We investigated many data fusion studies dedicated to IoT by considering two perspectives: (1) the data fusion method, and (2) the data fusion level. From the data fusion methods perspective, Figure 1 shows a mapping of data fusion approaches for different IoT domains based on data fusion methods [12]. The main trends include: (1) probability-based methods (PBMs) that express the relationships among diverse variables using estimation methods, i.e., Bayesian inference and Kalman filtering, but mainly suffer from limited performance when dealing with complex and multivariate data; (2) evidence reasoning-based methods (EBMs) that introduce the concepts of belief and plausibility to show uncertainty in a given situation, i.e., Dempster–Shafer (D–S) theory and recursive operators, but encounter difficulty in estimating mass functions with unreliable data; and (3) knowledge-based methods (KBMs) which gain knowledge from huge data, including intelligent aggregation methods, such as supervised learning, i.e., k-nearest neighbor, support vector machines, naive Bayes, artificial neural networks and unsupervised learning, i.e., k-means [13]. 

From the data fusion level perspective, various data fusion methods have been considered for IoT systems at different data fusion levels. Most of these methods consider domain-specific approaches, in which they adopt either data in–feature out, feature in– feature out, feature in–decision out or decision in–decision out perspectives to provide domain-specific applications. However, the scope of this study includes fusing data directly from IoT sources at a low-level data perspective, adopting the data in–data out level, handling challengeable IoT data features to achieve further business purposes based on the IoT domain, despite the expected outcome after fusion being data, features or decisions. Accordingly, this section investigates the main low-level data fusion approaches that have been proposed for IoT-based systems and evaluates them from the perspectives of both big data characteristics and IoT data features to highlight the current research gaps in this area. The authors of [12] introduced a healthcare data fusion model on the cloud for complex health event detection by aggregating individual elements over multiple data sources using the complex event processing (CEP) method. A main concern in this model was that it ignored the mobility of IoT data while fusing healthcare data. Moreover, it did not address the data expiry intervals used for analysis. In [13], an anomaly detection data fusion approach on the cloud was proposed to detect misbehaviors and inaccurate data source readings for robot motion. Inaccurate actuator and corrupted sensor readings were determined using a model-based estimation data fusion method. Neither the data source trustworthiness nor timely IoT data fusion at a certain time/event and sensor metadata were considered during the analysis, which affected the accuracy of the analysis results.

Another data fusion model for IoT was introduced in [14] for real-time energy pricing using the distributed state estimation method, aggregating data from various power data sources. However, it did not consider a data expiry interval in the real-time analysis. A hierarchal data fusion model for gesture recognition imagery was proposed in [15]. It maintained and reduced the dimensions of images using principle component analysis (PCA), and then fusion started by splitting images based on color and information depth using Gaussian model segmentation and a recognition process using SVM classification. The model neither addressed the image metadata nor considered the expiry of the images, affecting the analysis accuracy. Moreover, the model had a negative processing performance due to the central processing. 

An optimized model for sensor data allocation was introduced in [16] using the particle swarm optimization (PSO) data fusion method, considering sensors’ spatial information. However, it did not handle the sensors’ metadata and temporal data during the analysis. Another human transition detection model was proposed in [17] based on fusing captured images and body-deployed sensors using convolutional neural networks (CNN) for transition movements and fall detection. This model ignored the real-time processing of the big data veracity, the semantic information included in the sensor metadata and the data expiry rate, which negatively impacted the model accuracy and performance. For smart environments, the authors in [18] introduced a weather forecasting data fusion model using the kriging with external drift (KED) method. Although several IoT data features were considered in this proposed model, it did not manage inaccurate sensor data. 

A data fusion approach for nuclear power crack detection was proposed in [19] using visual-based data. First, CNNs were used to detect crack patches, and NB ensured whether the detected cracks were real or not. Yet, the approach ignored the metadata and data accuracy during the analysis, which affected the analytical accuracy. Another data fusion model for opinion mining in social networks was introduced in [20]. The model started by filtering the unrelated data and then grouping the similar data using k-means clustering. It considered neither opinion expiry rates nor opinion temporal features during the analysis, presenting a serious concern. Moreover, using central processing for analysis resulted in a poor processing performance.

For the wireless communication field, a data fusion model for automatic modulation recognition (AMR) was proposed in [21] using the global average and max pooling (GAMP) method. The fusion model was responsible for extracting relevancy between spatial and temporal signal features. However, the model ignored the data expiry rate and signal source trustworthiness, which downgraded the result accuracy. Another video summarization data fusion model was proposed in [22] on the cloud to enhance the video retrieval process. The proposed model used CNNs to extract video features while preserving big data characteristics. Yet, it did not preserve video-associated metadata during the analysis.

Considering all the presented studies, IoT has become a major source of big data. Therefore, managing big data complexity is essential while processing IoT data. This can be achieved by reducing the big data volume, supporting the processing of diverse data types and ensuring real-time processing of the data velocity rather than using a traditional central processing approach. However, many data fusion approaches do not consider such issues during analysis, as discussed earlier. Another major concern is that all the studies presented data in–decision out data fusion approaches, which makes them domain-specific and data-dependent approaches with poor adaptability to different domains. Table 1 presents a comprehensive summary of the investigated data fusion approaches, associated with their evaluation from the perspectives of big data characteristics and IoT data. 

## 3. Problem Definition and Main Contributions

Data fusion for IoT concerns all data types to be collected, transmitted, aggregated from different sources and processed for better decision making. New data features related to the IoT domain can be inferred as per the discussed previous studies, which added further complexity to the data fusion process. Although many studies have tried to customize their data fusion approaches for IoT, most of them were application specific for certain analytical purposes, as they restricted their data fusion outcome to be domain-dependent features or application-dependent decisions. Hence, this section elaborates the deduced IoT data features and discusses their impact on the current data fusion approaches for IoT systems, followed by our main contributions in the proposed STDF.

### 3.1. IoT Data Features

**Figure 2:**
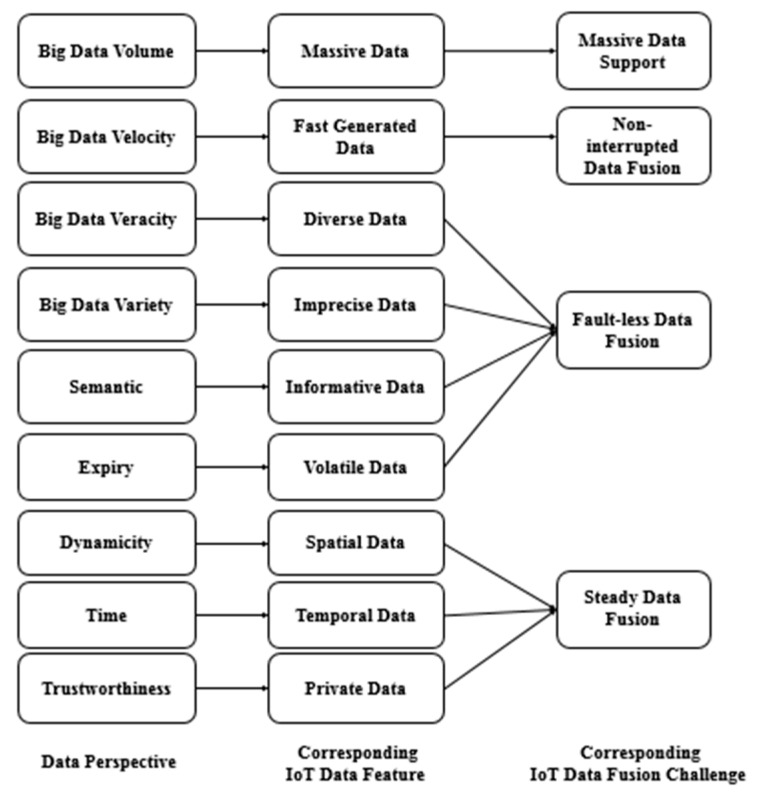
The inferred IoT data features and their corresponding processing challenges.

Specifying the unique characteristics of IoT data helps to determine the requirements of data fusion to consider for IoT systems. Figure 2 summarizes the explored IoT data features with respect to the IoT data perspectives investigated in Section 2 across different IoT domains, mapping them to their resultant processing challenges. These features should be specifically addressed in IoT-related data fusion approaches. A detailed explanation is presented below.

#### 3.1.1. Common Features of Big Data Characteristics

IoT systems are concerned with a large number of sensors that continuously observe physical objects and generate big data. As the technology advances, more characteristics are added to big data [23]. However, data fusion for IoT systems should maintain the following big data characteristics related to the IoT:

- Massive data: this feature is related to the big data volume, which expresses the huge amount of sensed and actuated data that need powerful processing techniques;
- Fast generated data: this feature relates to the big data velocity, in which the fast data generated require continuous processing to cope with the high data speed;
- Diverse data: this feature reflects the big data variety, in terms of the diversity and heterogeneity of the collected data types and structures;
- Imprecise data: this is concerned with the big data veracity, in terms of data imperfection, conflict, ambiguity and inconsistency due to perceiving data by various sensors.

#### 3.1.2. IoT-Specific Data Features

- Informative data: IoT data are associated with valuable metadata, enriching data with more information and semantics. This reveals a new factor for data fusion accuracy.
- Volatile data: This indicates either the data freshness or expiration to be used in analytics. For example, traffic data must be refreshed at short time intervals otherwise they will be useless. In contrast, environmental data for the same area could be used for longer periods. This feature directly impacts the quality of data fusion.
- Spatial data: In some IoT domains, IoT data are location based, which indicates they are dynamically changing and spatially correlated.
- Temporal data: Considering the time factor, this reflects whether the data are constant or time variant. In some domains, IoT data are time based that need regular processing and analysis as per specific times, e.g., in the renewable energy domain, tidal power data analysis needs to extract data only at tidal time periods instead of continuously extracting data which is a useless process.
- Private data: In some IoT domains, IoT data can be collected and accessible from public sources. This often requires feeding more information to trust these data sources.

### 3.2. IoT Data Processing Open Issues

Considering the data fusion approaches in IoT-based systems and inferred IoT-related data features discussed earlier, many processing challenges of IoT data have been ignored. Figure 2 presents our deduced IoT data fusion processing challenges and their relation to our inferred IoT data features, as explained below.

#### 3.2.1. Massive Data Support

The massive amounts of generated IoT data consume excessive processing resources and storage, downgrading the overall processing performance using limited resources or central processing techniques. Data fusion should be efficient and scalable to process different data sizes. Otherwise, it would aggravate the imperfection of the collected data and produce incorrect results. This problem arises as a result of ignoring the massive IoT data feature.

#### 3.2.2. Non-Interrupted Data Fusion

Efficient data fusion supports fast responses and continuous processing to ensure sensors collect data for fusion without causing bottlenecks and latency in IoT services. Discarding the fast generated data feature while obtaining IoT data using either offline or complex processing operations decelerates the overall system response.

#### 3.2.3. Fault-Less Data Fusion

Accurate data fusion results are directly related to data extraction. Therefore, handling IoT data inconsistencies, noise and heterogeneity is required during data acquisition. During processing, IoT metadata highly enrich the obtained information, which improves the data fusion result accuracy. Moreover, maintaining the data freshness ensures accurate results. Therefore, the data fusion accuracy is affected by ignoring imprecise, diverse, informative and volatile data.

#### 3.2.4. Steady Data Fusion

Data fusion results determine a specified decision, such as a diagnosis or emergency response. Unreliable fusion results may cause an intolerable danger. Thus, reliability is a basic requirement of data fusion approaches that is directly affected by spatial and temporal data, in which decisions may vary as data vary. In addition, ensuring the trustworthiness of IoT data sources before data extraction is vital for data fusion reliability.

### 3.3. The Main Contributions

Data fusion has been efficiently applied to reconstruct data, conduct predictions and detect data anomalies. However, the deduced characteristics of IoT data introduce new challenges for data fusion in IoT systems that would hinder the benefits of data fusion in such expanding systems. Thus, we propose the spatiotemporal data fusion (STDF) model that maintains the newly inferred IoT data features highlighted in Section 3.1, considering the IoT data processing challenges emphasized in Section 3.2. Thus, the main contributions of this study are aggregated in the proposed STDF model to provide a customizable IoT data fusion approach for IoT-based systems with the following characteristics.

#### 3.3.1. Domain-Independent and Spatial-Related IoT Data Fusion

To the best of our knowledge, STDF is the first DAI–DAO data fusion approach for IoT data that is independent of the data structure or the business analytics purpose. Hence, it can be integrated on the top of any IoT data source layer. STDF maintains the spatial IoT data feature by considering data locations as the main factor for data aggregation in the fusion process, which ensures accurate and corelated data fusion results.

#### 3.3.2. Temporal and Renewed IoT Data Fusion

For fast generated data in real-time systems, STDF considers the temporal IoT data feature by triggering the fusion process on specific events, which provides a reliable data fusion process for emergency reactions. STDF also maintains the volatile IoT data feature by ignoring expired data during data acquisition and fusion, ensuring accurate and updated data fusion results.

#### 3.3.3. Trusted and Scalable IoT Data Fusion

STDF preserves the IoT data trustworthiness via validating data sources during data acquisition for a steady and safe data fusion. STDF considers the double-stage cluster sampling technique for data reduction before data fusion to maintain the massive IoT data. Applying such data reduction on real-time stream processing would avoid processing bottlenecks and maintain the data fusion scalability and latency issues.

#### 3.3.4. Accurate and Real-Time IoT Data Fusion

STDF handles IoT data semantics and accuracy by considering metadata during data acquisition, which reveals data defects and adds more information for an accurate fusion. STDF is built on top of the “IoTSim-Stream” as an IoT-based stream processing simulator [24,25]. This provides a real-time processing infrastructure for continuous processing of fast generated IoT data, and for handling data fusion latency.

## 4. The Proposed Spatiotemporal Data Fusion (STDF) Approach

**Figure 3:**
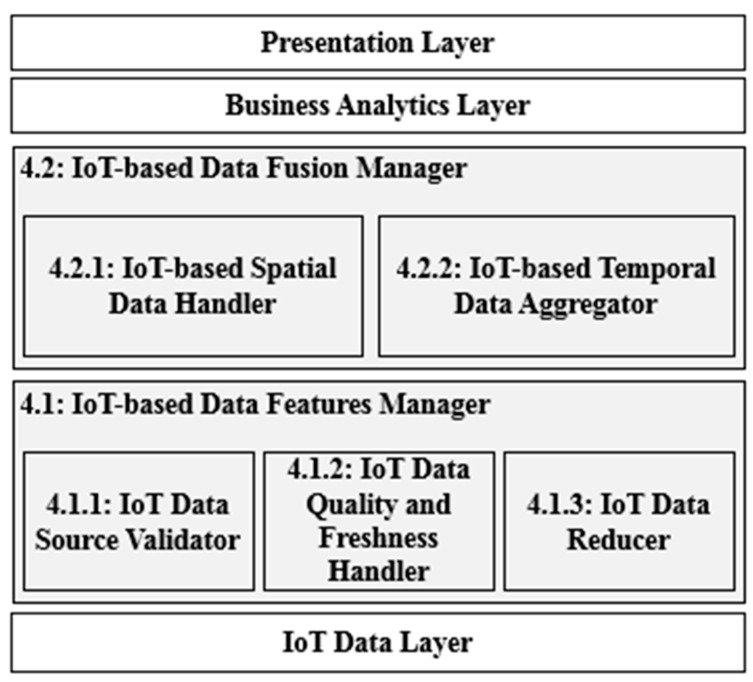
The proposed STDF architecture.

In this section, we present a detailed explanation of the proposed IoT-based spatiotemporal data fusion (STDF) approach. The STDF functionalities can be categorized mainly into two layers dedicated to handling the deduced IoT-specific data features: (i) the IoT-based Data Features Manager, which is responsible for considering most of the IoT data perspectives in the low-level sensor data, and (ii) the IoT-based Data Fusion Manager, which resides in a remote server where the data fusion process is accomplished. Figure 3 shows the proposed architecture of STDF, which shows how the two layers of the proposed STDF reside among the IoT data layer, the business analytics layer and the presentation layer of any domain-specific IoT application.

The IoT data layer is responsible for connecting the IoT physical data sources for real-time data acquisition. This can include any IoT data sources of any domain. On the other side, the business analytics layer performs the required analytics to generate application-specific decisions based on the defined business requirements of the IoT system after fusing IoT data and maintaining their IoT data features to ensure the quality of the fused data. The presentation layer is responsible for presenting the analytical results to the system users, in which various presentation tools can be plugged in as per the system needs.

In this context, the IoT data layer, the business analytics later and the presentation layer are out of the scope of the proposed STDF approach, in which all are completely independent, application specific and related to the domain of the IoT-based system in hand and, thus, do not affect the processing of STDF. STDF can be integrated with any IoT-based system to provide efficient IoT-oriented DAI–DAO fusion capability for any IoT data sources to be represented in any analytical business logic for further decision making purposes. Hence, STDF can be deployed in any distributed, cluster-based streaming framework having the capabilities of spatial partitioning (e.g., GeoHash), DAG operator placement and processing or message middleware solutions such as Kafka and NATS [26,27].

Accordingly, STDF assumes that all data sources at the IoT data layer generate the same data structure. Thus, the data in STDF are manipulated as data packets/units. For example, geographic data sources might generate imagery data, while healthcare data sources might generate numeric and imagery data. In the following sub-sections, a detailed clarification of the STDF layers and their modules is discussed.

### 4.1. IoT-Based Data Features Manager

This layer directly operates on the top of the IoT data layer where raw data are continuously received from different IoT devices to maintain the IoT data trustworthiness, data expiry and volume. The metadata should be kept for each received data unit such as the source ID (Source ID), generation time (GT), data unit size and location ID (LocID). This layer includes three sub-modules as follows. 

#### 4.1.1. IoT Data Source Validator

This module ensures the trustworthiness of the IoT data by calculating the trust degree of each Source ID to validate it. Each IoT source is identified by several parameters such as the energy rate, as most IoT sources operate at a low energy rate (in watts), and the processing size and storage size (in megabytes), as most IoT sources have minimal processing and storage capabilities [28]. Let $WT_{i}$ be a user-defined weight based on the IoT domain assigned to each parameter i, and let $PR_{ij}$ be the value of parameter i at data source j. The weight values are fixed for all sources and in a range of 0–1, in which the summation of all weight values equals 1. STDF ensures the trustworthiness of each IoT source j by calculating its trust degree $T_{j}$, as shown in (1) [29]:

(1) $$T_{j}=\overset{i=n}{\underset{i=1}{\sum }}WT_{i\;}\cdot PR_{ij}$$

where $WT_{i}$ is the weight value of the parameter i, $PR_{ij}$ is the value of parameter i at source j and n is the total number of considered parameters. After calculating the trust degree $T_{j}$ for each source j, STDF prevents the ignored source $IS_{j}$ that has a trust degree $T_{j}$ greater than or equal to a user-defined trust degree threshold TR, as shown in (2):

(2) $$IS_{j}=T_{j}\geq TR\;$$

where $IS_{j}$ represents the ignored source j, $T_{j}$ is the trust degree value of source j and TR is the user-defined trust threshold.

#### 4.1.2. IoT Data Quality and Freshness Handler

The faults of IoT actuators are crucial in any IoT domain. Thus, managing the corruption and loss of sensor data to ensure the data quality is essential before any further processing. Accordingly, STDF initially groups data based on their source. It then separately maintains the data per group to detect outliers using the statistical window-based approach “low–high-pass filter”, which classifies the data as faults or anomalies based on a user-defined interval dependent on the IoT domain [30]. For each group, STDF then replaces the missing data and detected outliers per attribute i with the mean value of the attribute $M_{i}$. For instance, if there are x attributes, STDF calculates $\left(M_{1},\;M_{2},\ldots ,\;M_{i},\ldots M_{x}\right)$, as shown in (3) [31]:

(3) $$M_{i}=\frac{1}{n}\overset{j=n}{\underset{j=1}{\sum }}y_{ji}$$

where $M_{i}$ is the mean value of only the valid data units of attribute i, n is the number of only the valid data unit measurements of attribute i and $y_{ij}$ is the attribute measurement at data unit j of attribute i. Moreover, volatile data are a domain-specific feature. For example, traffic data are fresh for seconds, while geographic data are fresh for months or longer. Thus, this module handles the data expiry perspective of IoT data by checking that the generation time of a data unit belongs to a specific time frame specified for each domain. 

#### 4.1.3. IoT Data Reducer

This module handles the big IoT data volume by reducing the number of data units used for fusion using the double-stage cluster sampling technique [30]. In this technique, the total population is divided into groups (clusters), and a random sample of all groups is selected rather than selecting the whole elements from one cluster. In this module, the data units are first clustered into groups based on their Source ID. 

Since STDF is a DAI–DAO domain-independent data fusion approach, all IoT sources are assumed to have the same importance. Instead of selecting all data units of one cluster, STDF samples each cluster by applying the probability proportional to size (PPS) sampling method, in which the probability of the selected data unit is proportional to the size of the cluster, meaning that larger clusters have a higher probability of selection and smaller clusters have a lower probability [32,33,34]. Since IoT data sources can generate a variable number of data units, the clusters’ sizes would be unequal. This contradicts the sampling concept, which assumes that all data units are equally likely to be chosen [35,36]. For example, if one cluster has 20,000 data units, the probability of a data unit being selected would be 1/20,000 (0.005%), while if another cluster has 10,000 data units, the chance of a data unit being selected would be 1/10,000 (0.01%). In order to deal with such differences, STDF retains the fixed sample size for each cluster by compensating the unequal probabilities of selection using weights. This ensures that all data units in the population have the same probability of selection irrespective of their cluster size. PPS guarantees the same weight value W over all clusters, which is calculated using (4) [37]:

(4) $$\;W=1/{(P}_{1}*P_{2})\;$$

where $P_{1}\;$is the probability of each sampled cluster, and $P_{2}$ is the probability of each sampled data unit, which are computed as shown in (5) and (6), respectively:

(5) $$P_{1}=(a*d)/b\;$$

(6) $$P_{2}=c/a\;\;$$

where a is the number of data units in the cluster, b is the total number of all data units, d is the number of clusters, which represents the number of sources of the data units in STDF, and c is the number of data units to sample in each cluster, which is a user input.

Algorithm 1 presents the pseudocode of the data reduction mechanism applied in STDF. This mechanism is performed directly after data acquisition and before submitting the data units to the processing server. It starts by checking both the data units’ SourceID and GT. Then, it groups the data units based on the same SourceID. Next, it calculates the sampling size by determining the sampling parameters ($P_{1}$, $P_{2}$ and W) for each group to sample it.

In case of contingencies, shortage scenarios and searching for fast recovery schemes, such as large-scale electrical power system recovery, each sensor should be analyzed. This would contradict the added value of using the IoT Data Reducer, as it might require taking every bit of data sent by the sensors for recovery purposes.
**Algorithm 1:** STDF IoT-based Data Features Manager.  Input: Arraylist ‘L’ of acquired data units  Output: HashMap ‘HC’ of key: source id and value: Arraylist of arraylist 1 2 3 4   5 6 7 8 9 10   11 12 13 14 15 16 17 18 19 20 21 22 23 24 25 26 27 28 29 30   31 32 33 34 35 36 37 38 39 40   41 42 43 44   45 46 47 48 49   50 51   52 53 54 55 56 57 58 59 60 61   62 63 64 65 **Begin**  Initialize DL as an empty Arraylist//initialize the list for the sample  **For each** data unit ‘D’ in L//loop for all received data units     **If** (IoT Data Source Validator (‘D’) = True and IoT Data Quality and Freshness Handler (‘D’)     = True) **then**//check if the source ID is valid and data unit is fresh  {Add ‘D’ in DL}//add this data unit to the list as a candidate  **End if**  **End for**  Initialize HC as an empty HashMap  **For each** data unit ‘D’ in DL//loop for all data units to group them based on their source ID     **If** (source ID of ‘D’ in HC keys) **then**//check if there is a key in HC equals the source ID      of the current data unit  {Add ‘D’ in HC key}//add the current data unit to this key  **Else**//create a new key in HC with the value of this source ID  {Set source ID of ‘D’ as a new key  Add ‘D’ in HC key}  **End if**  **End for**  **For each** key in HC//loop for all source IDs in HC to calculate mean value ‘M’ for each attribute  Initialize data_units_List with the current source ID’s data units  Initialize attributes_mean_List as an empty array list  **For each** attribute ‘AR’ in data_units_List//loop for all attributes  Initialize attribute_sum = 0//The summation of attribute’s values   Initialize data_units_count = 0//The count of data units  Initialize M = 0//Attribute’s mean value  **For each** data unit ‘D’ in AR//loop for all attribute’s values per each data unit  **If** (low–high pass filter = True) **then**//check if there is no outlier or missing value  {attribute_sum = attribute_sum + D  data_units_count = data_units_count + 1}  **End if**  **End for**  M = attribute_sum/data_units_count//divide the summation of attribute’s values by   the count of data units  Add M to attributes_mean_List  **End For**  **End for**  **For each** key in HC//loop for all source IDs to clean missing and outlier data units in each group  Initialize data_units_List with the current source ID’s data units  **For each** data unit ‘D’ in data_units_List//loop for all data units’ candidates  **For each** attribute ‘AR’ in D//loop for all attributes per each data unit  **Get attribute’s mean value** ‘M’ from attributes_mean_List  **If** (low–high pass filter = False) **then**//check if there is an outlier or missing value  {Replace AR of D with M}//Replace the outlier or missing attribute value in  the data unit by the attribute mean value   **End if**  **End for**  **End For**  Update the current source ID’s data units with data_units_List//update each source ID  corrupted data units with the cleansed data units  **End for**  Initialize population_counter = 0  Initialize sample_size = X  **For each** key in HC//loop for all source IDs in HC to calculate the population_counter over them     Add count of data units to population_counter//add the data units count of the current      source ID to population counter  **End for**  **For each** key in HC//loop for all source IDs in HC to perform sampling and create the attributes   mean vector as the state-estimator  Initialize data_units_List with the current source ID’s data units  Initialize MV as an empty Arraylist//initialize the attributes mean vector  **For each** attribute ‘AR’ in data_units_List  Calculate AM using all data units of the current source ID//obtain every attribute mean  Add AM in MV//add the attribute mean value to the attributes mean vector  **End for**     Calculate P1 using the population_counter and the data units of the current source ID      Calculate P2 using the sample size and data units of the current source ID     Calculate Sample weight using P1 and P2 of the current source ID     Apply sampling with PPS using the current sample weight and data units of the current      source ID//reduce the data units of the current source ID  **End for**  Return HC as a list of pairs (source ID, sampled data units, MV)//return a HashMap of   key: source ID and values: list of sampled data units and the attributes mean vector  **End**

Accordingly, sampling each cluster while ignoring the other clusters’ population could lead to serious problems. To handle such operational scenarios, the IoT Data Reducer in STDF uses a state estimator to estimate the unsampled data units. Since STDF uses the double-stage clustering technique, it first computes the first stage cluster mean vector $MV_{i}$ per cluster i as a state estimator [38]. For instance, if there are x clusters, STDF computes $\left(MV_{1},\;MV_{2},\;\ldots ,\;MV_{i},\ldots ,MV_{x}\right).$ STDF then attaches an $MV_{i}$ value with the sampled data units for cluster i to the processing server for further business purposes. $MV_{i}$, the mean vector of cluster i, represents all the attributes’ mean values $AM_{j}$, which is the mean value of attribute j that considers all data units in the sample after being cleansed in the IoT Data Quality and Freshness Handler, calculated as shown in (7) and (8). Let z be the number of attributes in the population,$\;MV_{i}=\left[AM_{1},\;AM_{2},\ldots ,AM_{j},\ldots ,AM_{z}\right]$.

(7) $$Pop=\begin{array}{cccc} d_{11} & d_{12} & \ldots & d_{1z}\\ d_{21} & d_{22} & \ldots & d_{2z}\\ d_{N1} & d_{N2} & \ldots & d_{Nz} \end{array}$$

(8) $$AM_{j}=\frac{1}{N}\overset{i=N}{\underset{i=1}{\sum }}d_{ij},\;j=1,\;\ldots ,\;z$$

where Pop represents the cluster population of N data units with z attributes in the cluster, and $AM_{j}$ is the mean value of attribute j.

### 4.2. IoT-Based Data Fusion Manager

This layer lies in the remote server where all the reduced data units resulting from the IoT-based Data Features Manager are received to manage the spatial and temporal data fusion process using the following modules.

#### 4.2.1. IoT-Based Spatial Data Handler

After receiving the reduced and grouped data units based on their SourceID, this module is responsible for handling the spatial feature of the IoT data. It clusters all data units based on their LocID using the k-means clustering technique [39]. STDF applies k-means to each group of data units generated from the same data source. Hence, the K centroids are set to be the location IDs of all data units of the same SourceID. Only one clustering iteration is needed to cluster the data units according to these fixed K centroids, since there is no need to update the centroids in this case, by calculating the Euclidean-based distance ${\mathrm{Ed}}_{i}$ between each data unit’s location ID DlocID and all centroids’ $K_{i}$ in ($K_{1}{,K}_{2}{,\ldots K}_{n}$), as shown in (9) [40]:

(9) $$Ed_{i}=\sqrt{{\left(DLocID-K_{i}\right)}^{2}}$$

Therefore, each data unit will have n distances with respect to the n centroids. However, the data unit is assigned to cluster $K_{i}$ having ${\mathrm{Ed}}_{i}$ = zero. This generates clusters of data units with the same LocID. In some IoT domains, the LocID identifier is not provided in a discrete number format, e.g., using the GPS system. In such cases, STDF discretizes the location coordinates by classifying n location IDs into n intervals with a domain-dependent user-defined cut point (upper and lower bound coordinates). STDF then calculates the Euclidean-based distance $D_{i}$ between each coordinate (x,y) and the *n* upper (or lower) bounds $\left(x_{1},y_{1}\right),\;\left(x_{2},y_{2}\right),\|\left(x_{n},y_{n}\right)$, as shown in (10). The bound with the lowest distance value will hold this coordinate. After discretizing all coordinates into discrete location IDs, STDF applies the k-means clustering technique [41].

(10) $$D_{i}=\sqrt{{\left(x-x_{i}\right)}^{2}+{\left(y-y_{i}\right)}^{2}}\;$$

Considering the k-means clustering consistency, it could be affected by several factors including: (1) random selection of centroids, (2) data quality and (3) huge data volume [42,43]. STDF ensures clustering consistency by setting fixed K centroids, managing data outliers and data loss via the IoT Data Quality and Freshness Handler and reducing data through the IoT Data Reducer. Thus, the clusters’ consistency is maintained.

#### 4.2.2. IoT-Based Temporal Data Aggregator

Following Dasarathy’s data fusion classification, this module adopts the DAI–DAO data fusion plan [44]. All data units of all source IDs are globally aggregated at the different location IDs by applying the Tiny AGgregation (TAG) data aggregation technique [45,46,47] on the resultant location-based clustered data units to obtain the freshest data units of the same SourceID at all visited locations. STDF utilizes TAG to perform a classic hierarchy-based aggregation process, which structures multiple trees of location IDs (leaves) per SourceID (root). The minimum Min over the GT is considered as a duplicate-insensitive aggregation function to query all data units ${(D}_{1}{,\;D}_{2}{,\;\ldots D}_{n})$ for each LocID and obtain the freshest data unit at this location ${\mathrm{FreshestD}}_{\mathrm{LocID}}$, as shown in (11):

(11) $$FreshestD_{locID}=\underset{1\leq i\leq n}{\min}(D_{i}.GT)$$

**Figure 4:**
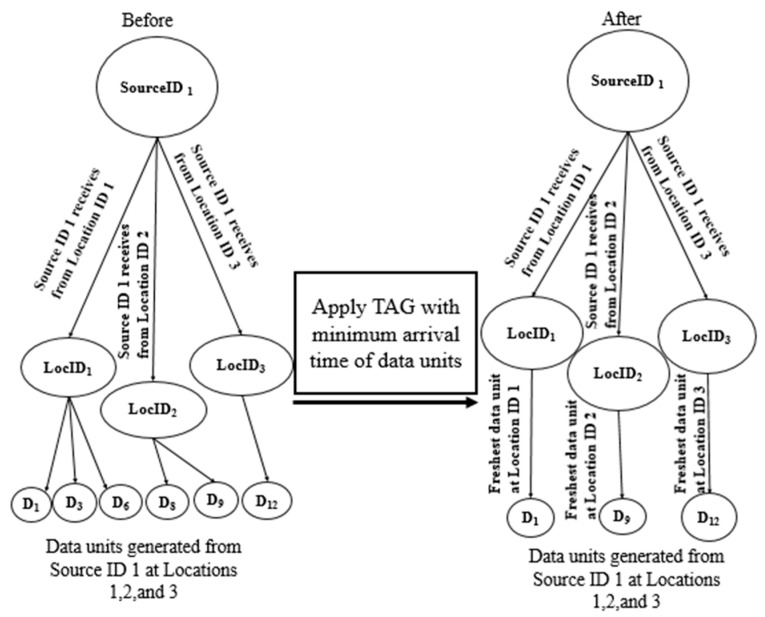
The TAG data aggregation technique in STDF.

Figure 4 illustrates the trees’ structure before and after STDF data fusion. For instance, before data fusion, ${\mathrm{SourceID}}_{1}$ is connected to ${\mathrm{LocID}}_{1}{,\;\mathrm{LocID}}_{2}$ and ${\mathrm{LocID}}_{3}$, where ($D_{1}{,\;D}_{3},$ $D_{6}$), ($D_{8}$, $D_{9}$) and $D_{12\;}$ are generated, respectively. After data fusion, considering the minimum GT of the data units at each location, ${\mathrm{SourceID}}_{1}$ keeps $D_{1}{,\;D}_{9}\;$ and *D*_12_ as the freshest data units at ${\mathrm{LocID}}_{1}{,\;\mathrm{LocID}}_{2}$ and ${\mathrm{LocID}}_{3}$, respectively. This module is temporal event triggered, in which the aggregation process fires in STDF when the event of receiving the location-based clustered multimodal data units occurs. Algorithm 2 presents the pseudocode of the whole spatiotemporal data fusion process. The data fusion process is performed at the processing side upon receiving the SourceID and its data units. Considering that the location IDs are fixed, one iteration of k-means clustering is applied per data unit of the same ID, in order to be clustered according to the location IDs (fixed K centroids) to minimize the processing time. Next, all source data units are globally aggregated at all location IDs using TAG with the minimum aggregation function over the GT of data units on an event basis to ensure data freshness.

**Figure 5:**
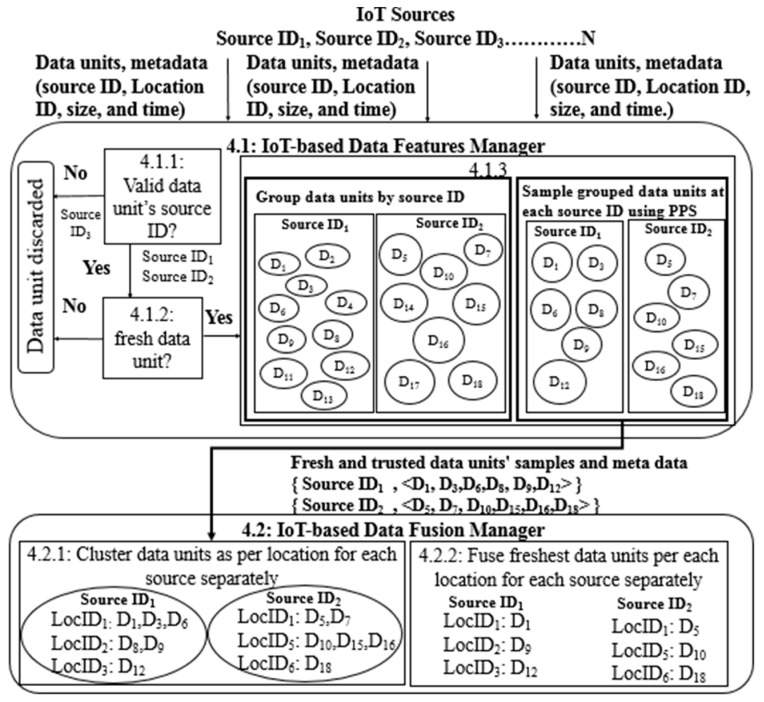
The proposed STDF processing scenario.

Figure 5 illustrates the complete processing scenario of STDF, which begins after data acquisition and ends before business analytics. The scenario assumes that several IoT data sources,${\;\mathrm{SourceID}}_{1}{,\;\mathrm{SourceID}}_{2}\;$and ${\mathrm{SourceID}}_{3}$ are continuously generating massive amounts of data associated with valuable metadata, which are transmitted to the IoT-based Data Features Manager. The data are acquired and checked for trustworthiness. For instance, the data units of ${\mathrm{SourceID}}_{3}$ are ignored because of the invalidity of this source as per Section 4.1.1, as well as the data freshness of the valid sources as per Section 4.1.2.
**Algorithm 2:** STDF IoT-based Data Fusion Manager.**Input:** HashMap ‘HC’ of key: source id and value: Arraylist of data units **Output:** HashMap ‘Final-HC’ of key: source id and value: 2D Arraylist of location ID & data unit 1 2 3 4 5 6 7   8 9 10 11   12 13   14 15 16 17 18 19 20 21 22 23 24   25 26 27 **Begin**  Initialize Clustered_HC as an empty HashMap//the HashMap to retain location IDs clustering results  **For each** key in HC//loop for all received source IDs   Initialize Centroids_List as an empty Arraylist  Initialize data_units_List with the current source ID’s data units  **For each** data unit ‘D’ in data_units_List//loop for all data units of the current source ID  **If** (location ID of ‘D’ is not in Centroids_List) then//check if the location ID of the current   data unit exists in the centroids list  {Add locationID of ‘D’ to Centroids_List}//add location ID if it does not exist}  **End if**  **End for**   Cluster with K-means using the current Centroids_List and data_units_List//apply clustering on    the current source ID’s data units using its current centroids   Add key to Clustered_HC//retain the current source ID in the HashMap   Add clustered data units to Clustered_HC//retain the resultant clustered data units for the    current source ID in the HashMap  **End for**  Initialize Final-HC as an empty HashMap//the HashMap used for data fusion  **For each** key in Clustered_HC//loop for all source IDs   Initialize ArrayList Updated-Tree as an empty Arraylist  Initialize ArrayList TAG-Tree with the clustered data units of the current source ID   **For each** locationID ‘loc’ in TAG-Tree//loop for all location IDs of current source ID  Initialize data_units_List with the data units of the current ‘loc’  Get the ‘Freshest data unit’ with the minimum generation time in data_units_list   Add the current ‘loc’ and its ‘Freshest data unit’ in Updated-Tree  **End for**   Add the current key and its Updated-Tree to Final-HC//retain the aggregated results on all location   IDs of the current source ID in the final HashMap   **End for**  Return Final-HC//return a HashMap of key: source ID and values: location IDs and freshest data units  **End**

Next, the freshest data are reduced as per their trusted source IDs as in Section 4.1.3 by grouping the data units and sampling each group separately. For example, the sampled data units at ${\mathrm{SourceID}}_{1}\;$are${\;D}_{1}$,${\;D}_{3}{,\;D}_{6}{,\;D}_{8}{,\;D}_{9}\;$and $D_{12}$, and they are $D_{5}{,\;D}_{7}{,\;D}_{10}{,\;D}_{15}{,\;D}_{16}{\;\mathrm{and}\;D}_{18\;}$at${\;\mathrm{SourceID}}_{2}$. The sampled data units are then transmitted to the remote processing server, where the IoT-based Data Fusion Manager globally identifies all location IDs for each source and clusters the received data units per location ID for each source as in Section 4.2.1. For example, the identified location IDs for ${\mathrm{SourceID}}_{1}$ are ${\mathrm{LocID}}_{1}{,\;\mathrm{LocID}}_{2}$ and ${\mathrm{LocID}}_{3}$, while ${\mathrm{SourceID}}_{2}\;$ is connected to ${\mathrm{LocID}}_{1}{,\;\mathrm{LocID}}_{5}\;$ and ${\mathrm{LocID}}_{6}$. After clustering the sampled fresh data units at the trusted ${\mathrm{SourceID}}_{1}$, ${\mathrm{LocID}}_{1}{,\;\mathrm{LocID}}_{2}\;$ and ${\mathrm{LocID}}_{3}$ contain ($D_{1}{,\;D}_{3}{,\;D}_{6}$), ($D_{8}{,\;D}_{9}$) and $D_{12}$, respectively. Finally, STDF aggregates all data units at all sources from different locations as per their GT, preserving the data freshness as in Section 4.2.2, where $D_{1}{,\;D}_{9}\;$and $D_{12}$ are the freshest data units from ${\mathrm{SourceID}}_{1}$ at ${\mathrm{LocID}}_{1}{,\;\mathrm{LocID}}_{2}$ and ${\mathrm{LocID}}_{3}$, respectively, while $D_{5}{,\;D}_{10}$ and $D_{18}\;$are the freshest data units from ${\mathrm{SourceID}}_{2}$ at ${\mathrm{LocID}}_{1}{,\;\mathrm{LocID}}_{5}\;$and ${\mathrm{LocID}}_{6}.$

## 5. The Experimental Evaluation

This section presents the experiments conducted to evaluate the layers of the proposed STDF approach: the IoT-based Data Features Manager and the IoT-based Data Fusion Manager, with respect to their performance. The following sub-sections present a detailed description of the experimental environment, the experimental case study and the used dataset, as well as the experimental results for both the IoT-based Data Features Manager and IoT-based Data Fusion Manager modules to demonstrate their appropriateness for IoT data processing from the processing time and accuracy perspectives.

### 5.1. IoTSim-Stream Simulator

The STDF approach has been developed on top of the “IoTSim-Stream” simulator, providing a real-time IoT environment [24,48,49]. IoTSim-Stream is an IoT simulator for real-time processing of big data that offers an environment to model stream graph applications in a cloud environment. The IoTSim-Stream simulator leverages the features of CloudSim by integrating stream processing with workflow scheduling and execution to execute stream graph applications (SGAs) in the cloud environment, as well as the features of different scalable (IoT) stream processing solutions and distributed cluster-based stream processing frameworks [26,27], e.g., spatial partitioning. It supports stream processing (via IoT-Stream simulation cloud resources and IoT-Stream simulation virtual machine services) and graph spatial partitioning (via the IoT-Stream simulation service layer). An SGA is composed of different IoT sources named “services” that initiate data units with an unknown structure in real time as “streams”. Thus, each stream is identified by its:

- Processed size measured in megabytes (MB);
- Source ID SourceID (that generates the stream);
- Location ID locID (the service ID that transmits the stream);
- Generation time GT (the time when the stream is generated).

Each service generates and receives streams from neighbor services to transmit them to the cloud data center for processing, maintaining a fast IoT data velocity and preventing processing bottlenecks in the huge IoT data volume. Thus, numerous IoT sources are simulated to continuously generate massive data units, associated in real time with their metadata to be processed on a cloud server, which facilitates the tracking of the processing time at each layer. However, the simulator does not handle the IoT data features demonstrated in Section 3. Hence, integrating the two layers of the STDF approach with the simulator would offer a comprehensive IoT processing environment.

### 5.2. The Experimental Environment and Dataset

In this section, we present our experimental methodology and the associated cloud environment and simulation configurations to validate the efficiency of the STDF layers. The experiments were conducted on a machine with a Core i7, 2.70 GHz, 1T hard disk space and 8 GB RAM. To model the cloud environment for our experiments, we configured one cloud data center with 1000 hosts, where each host has 64 cores (PEs) and each PE has 1000 MIPS, and there is 144,000 MB RAM per host. Multiple virtual machines (VMs) were configured with 2000 MIPS, 8192 MB RAM and 1000 MB/s bandwidth. 

Prior to the simulation configuration, we defined three linear stream graph application files (DAG files) of 10 sources, 20 sources and 40 sources. Each source generates different amounts of data units per second as follows: 1606, 3212 and 6424 data units per second, respectively. STDF performs a DAI–DAO fusion irrespective the streams’ data structure to support a domain-independent IoT data fusion. By setting the simulation time to 15 s, we generated three datasets with different sizes: a small dataset (D1) with 24,090 data units, a medium dataset (D2) with 48,180 data units and a large dataset (D3) with 96,360 data units, with challengeable concerns, such as: containing noisy data, missing readings and outliers, as well as variable LocID formats, such as discrete location IDs and GPS location coordinates. These configurations are read by the IoTSim-Stream simulator during the initialization phase to simulate the given stream graph application file. 

For all services in the DAG files, the original processing scenario of the “IoTSim-Stream” simulator starts by generating streams and then transmitting a mixture of the generated streams and neighbor services’ streams (without a specific basis of stream selection) to be processed in the cloud data center. This scenario is repeated every second. 

**Figure 6:**
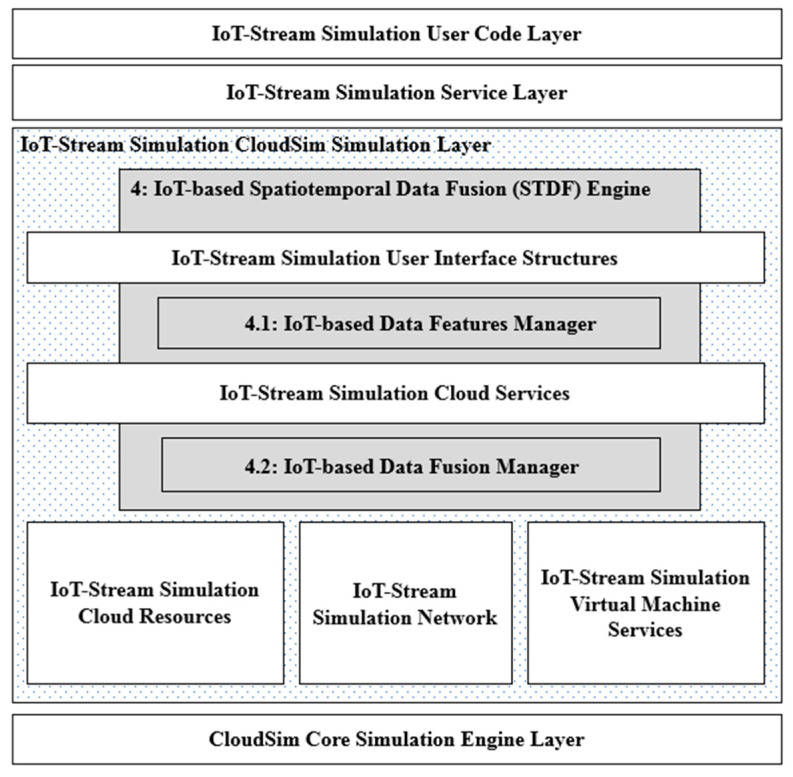
The proposed architecture of the spatiotemporal data fusion (STDF) engine on top of the IoTSim-Stream simulator.

Upon STDF integration, the IoT-based Data Features Manager receives a minor dataset every second to group and sample each SourceID with a specified number of streams and transmit them for processing at the server. Next, using the IoT-based Data Fusion Manager, instead of the random stream processing in the cloud data center in the original scenario, all streams of each SourceID are clustered per the same location ID where they are transmitted. For example, 80 streams of SourceID = 1 are clustered as 65 streams transmitted from loc ID = 1, 10 streams transmitted from loc ID = 2 and 5 streams transmitted from loc ID = 3. Then, a tree of clustered streams per location ID is built for each SourceID to aggregate the freshest stream (i.e., the stream with the minimum GT) per location. This scenario is repeated every second, fusing each SourceID fresh stream together from all transmitting location IDs. Figure 6 presents a modified system architecture of IoTSim-Stream, integrated with the STDF layers. The IoT-based Data Features Manager layer is added on top of the “IoT-Stream Simulation User Interface Structures” layer, where the SGA is parsed from the DAG file and the data units are acquired, whereas the IoT-based Data Fusion Manager layer is added to the “IoT-Stream Simulation Cloud Services” layer, where the data units are processed in the cloud server.

### 5.3. IoT-Based Data Features Manager Evaluation

This section discusses the performance evaluation of each module in the IoT-based Data Features Manager layer through the following investigations:

1. Validating the source IDs’ trust degree via the IoT Data Source Validator to evaluate the trustworthiness of STDF, ensuring a steady and secure IoT data fusion;
2. Checking the freshness of each stream via the IoT Data Quality and Freshness Handler, in order to manipulate the fault-less IoT data fusion challenge;
3. Ensuring the data stream quality by handling the data’s missing readings and outliers;
4. Checking the scalability of the proposed STDF IoT data fusion approach by reducing the amount of streams in the dataset before being transmitted to the cloud server via the IoT Data Reducer;
5. Checking the accuracy of the IoT Data Reducer for the fault-less IoT data fusion challenge;
6. Checking the processing time of the IoT Data Reducer to maintain a non-interrupted data fusion.

#### 5.3.1. Evaluating the Trustworthiness of STDF IoT Data Fusion

An experiment was performed to validate the IoT data sources by comparing each service’s trust degree $T_{j}$ to a certain trust threshold TR over D1. For instance, each service is assumed to have three parameters to consider: the energy rate in watts ($PR_{1j}$), the processing size in MB ($PR_{2j}$) and the storage size in MB ($PR_{3j}$). Three user-defined values of 0.2, 0.4 and 0.4 are assigned for the weights $WT_{1},\;WT_{2},\;and\;WT_{3}$ respectively. Table 2 presents the detailed values of $PR_{ij}$,$\;\;WT_{i}$ and $T_{j}$ for each service j. By setting a trust degree threshold of TR = 1200, $Service_{1}$ will be excluded to be $IS_{j}$, and therefore, all of its streams will be ignored in the entire data fusion process.

#### 5.3.2. Evaluating the Freshness of STDF IoT Data Fusion

**Figure 7:**
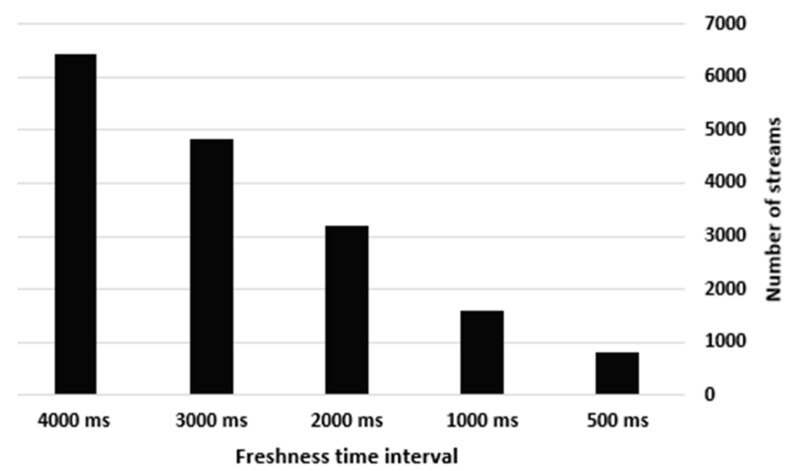
The number of fresh streams with respect to the different freshness time intervals.

After validating all the streams, the experiment in the IoT Data Quality and Freshness Handler tracks the number of fresh streams passed by their GT  using different time intervals. Hence, the number of fresh streams in D1 (before entering the IoT Data Reducer) was investigated at the following time intervals: 500 milliseconds (ms), 1000 ms, 2000 ms, 3000 ms and 4000 ms. As presented in Figure 7, the number of fresh streams that passed at the five predefined time intervals was as follows: 803, 1606, 3212, 4818 and 6424, respectively, which demonstrates that the number of fresh streams decreases by 50% when the freshness time interval decreases by 0.5 s, whereas it increases to 100% when the freshness time interval increases by 1 s. We adopted a freshness time interval of 1000 ms for all experiments, as the simulator generates and processes streams every second.

#### 5.3.3. Handling IoT Data Quality

The experiment in the IoT Data Quality and Freshness Handler shows how STDF handles the data’s missing readings and outliers by tracking the processed size generated per stream, i.e., from ${\mathrm{Service}}_{1}$ in D1. ${\mathrm{Service}}_{1}$ generates 139 streams with a processed size of a minimum of 50 MB and a maximum of 380 MB. However, by applying the low–high-pass filter on ${\mathrm{Service}}_{1}$ streams, only 130 streams passed, and 9 corrupted streams were found. Therefore, STDF replaced the 9 corrupted streams’ processed size with the mean value of the processed size of 327 MB as a result of adding the processed size of the remaining 130 streams/130.

#### 5.3.4. Evaluating the Reduction Scalability of STDF IoT Data Fusion

The IoT Data Reducer aims to manage the massive IoT data by reducing the generated streams using PPS sampling. Thus, this experiment demonstrates the resultant number of streams per second after applying PPS sampling at three fixed sample sizes of streams: 80, 100 and 120, for the three datasets (since one service generates between 135 and 175 streams) and a fixed freshness interval of 1000 ms. Table 3 illustrates the applied PPS approach over D1 per second using a sample size of 80. This approach ensures the same sampling weight over all the services (clusters) despite their different sizes.

**Figure 8:**
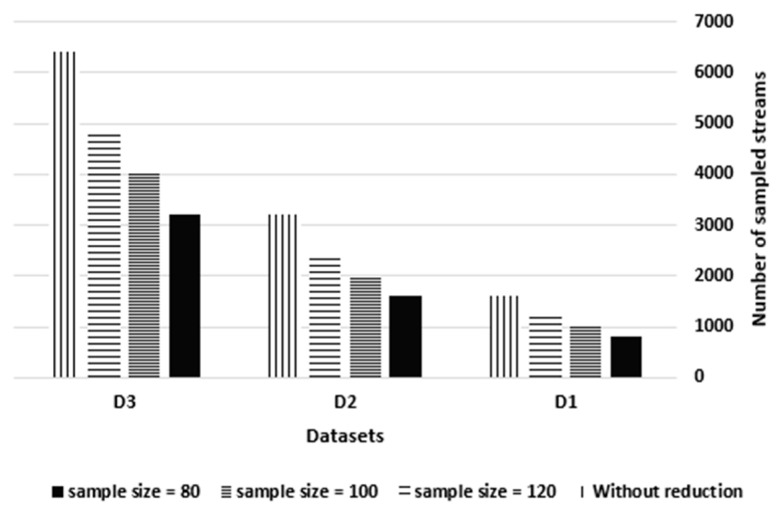
The number of sampled streams vs. the sample size.

As shown in Figure 8, the generated streams per second before sampling were 1606, 3212 and 6424 for the three datasets, D1, D2 and D3 respectively. At a sample size of 80, 800, 1600 and 3200 streams were generated per second for D1, D2 and D3, respectively. At a sample size of 100, 1000, 2000 and 4000 streams were generated per second, whereas at a sample size of 120, 1200, 2400 and 4800 streams were generated per second. This indicates that as the sample size increases by 25%, the sampled streams increase by 25%, reflecting the proportional relationship between the sample size and the number of sampled streams.

In case of contingencies, STDF attaches the attributes’ mean vector as a state estimator to the data units’ samples. Table 3 presents the attributes’ mean vector per service (cluster). Each stream is identified by the processed size, SourceID, LocID and GT. Only the processed size is an ordinal attribute; thus, the attributes’ mean vector equals the processed size mean as no mean value exists for the nominal attributes SourceID, LocID and GT [38]. STDF calculates the processed size mean per service by adding all service streams’ (both sampled and unsampled) processed size values and then dividing them by the service streams’ count, as shown in (7) and (8). Thus, the processed size mean was 327, 287, 291, 312, 278, 264, 274, 298, 277 and 280 MB for services 1, 2, 3, 4, 5, 6, 7, 8, 9 and 10, respectively.

#### 5.3.5. Evaluating the Reduction Accuracy of STDF IoT Data Fusion

After sampling the streams, this experiment investigated the accuracy of the resultant samples for an accurate data fusion performance using the variance Var, as shown in (12) [50]:

(12) $$Var=\frac{1}{n\left(n-1\right)}\times \overset{n}{\underset{i=1}{\sum }}{\left(y_{i}-Mpps\right)}^{2}$$

where n is the number of services per dataset, fixed as 10, 20 and 40 in our experimentation, $y_{i}$ is the sample mean of service i, which is calculated as per (13), and Mpps is the population mean of all services in the dataset, which is calculated as per (14).

**Figure 9:**
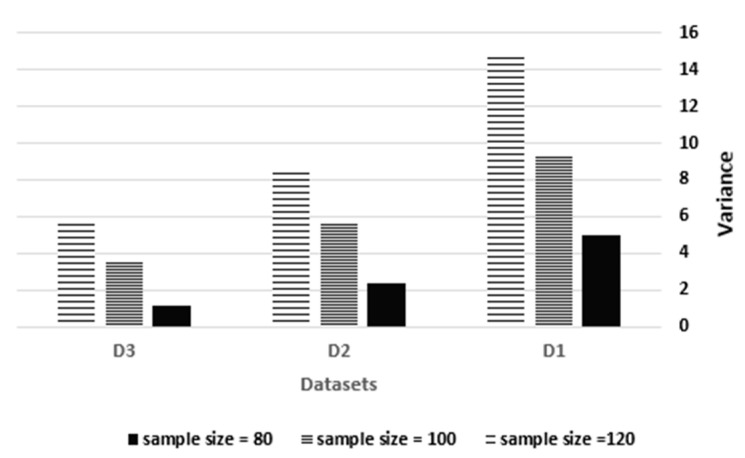
STDF sample variance vs. the sample size.

(13) yi=1m ∑j=1myj

where m is the number of sampled streams selected from service i, fixed as the three sample sizes of 80, 100 and 120 in our experimentation, and $y_{j}$ is the processed size value of stream j in service i.

(14) $$Mpps=\left(\frac{1}{n}\times \overset{n}{\underset{i=1}{\sum }}y_{i}\right)$$

where n is the number of services per dataset, and $y_{i}$ is the sample mean of service i. As presented in Figure 9, a sample size of 80 leads to variance values of 4.956, 2.348 and 1.144 over D1, D2 and D3 respectively, while a sample size of 100 leads to variance values of 9.344, 5.67 and 3.484, and a sample size of 120 leads to variance values of 14.682, 8.472 and 5.728. Therefore, the proportional relationship between the sample size and variance describes that an increase in the sample size by 25% will increase the sample variance by an average of 80%. By keeping the sample size constant, the reciprocal relationship between the dataset size and sample variance proves that increasing the dataset size by 100% decreases the sample variance by an average of 50%. 

#### 5.3.6. Evaluating the Real-Time Reduction Processing of STDF IoT Data Fusion

**Figure 10:**
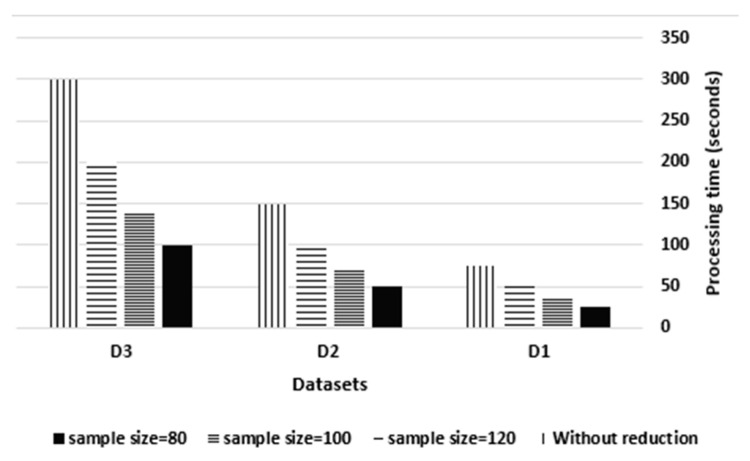
STDF processing time vs. the sample size.

This experiment tracked the simulator’s processing time in the cloud data center before and after sampling the streams for the three datasets to examine the real-time and non-interrupted processing of STDF. As shown in Figure 10, the processing time before sampling was 75, 150 and 300 s for D1, D2 and D3 respectively. Upon reduction at a sample size of 80, the processing time was 25, 50 and 100 s for D1, D2 and D3, respectively. At a sample size of 100, the time was 35, 70 and 140 s, while at a sample size of 120, the time was 45, 90 and 180 s. This reveals the proportional relationship between the sample size and the processing time, in which an increase in the sample size by 25% causes an increase in the processing time by 40%.

### 5.4. IoT-Based Data Fusion Manager Evaluation

This section evaluates the IoT data fusion scenario of STDF by examining each module in the IoT-based Data Fusion Manager layer through the following investigations:

1. Clustering streams of the same source ID to maintain their spatial feature via the IoT-based Spatial Data Handler;
2. Maintaining a massive IoT data fusion by aggregating the freshest streams per the location ID of each source ID via the IoT-based Temporal Data Aggregator;
3. Preserving both spatial and temporal IoT data fusion by tracking the performance of both the IoT-based Spatial Data Handler and IoT-based Temporal Data Aggregator over time, which ensures a fault-less and steady IoT data fusion;
4. Checking the processing time of the IoT-based Temporal Data Aggregator to ensure a non-interrupted IoT data fusion.

#### 5.4.1. Evaluating the Spatiality of STDF IoT Data Fusion

**Figure 11:**
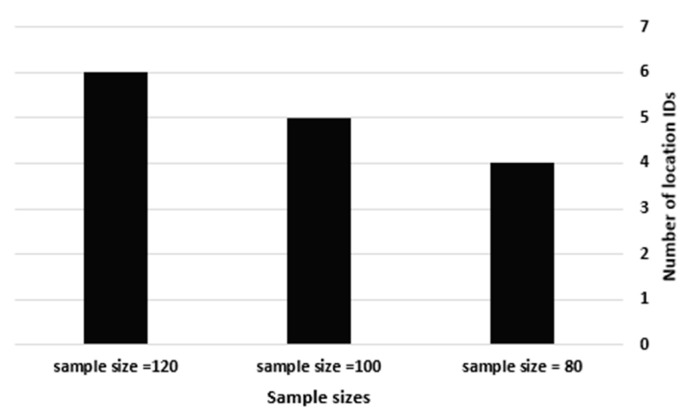
The number of location IDs vs. the sample size.

This experiment examined the IoT-based Spatial Data Handler to present the difference in the location IDs of specific SourceIDs through different sample sizes. Thus, we tracked the location IDs of ${service}_{1}$’s streams using sample sizes of 80, 100 and 120 over D1. There is no need to perform the same experiment over D2 and D3, as it is a factor of the sample size only. Hence, increasing the number of streams requires extra services to transmit them to the cloud data center. Table 4 shows how the sampled streams at a sample size of 80 are transmitted from four location IDs, while the streams generated at a sample size of 100 are transmitted from five location IDs, and the streams at a sample size of 120 are transmitted from six location IDs. Figure 11 visually shows that an increase in the sample size by 25% increases the number of location IDs by an average of 25%.

In some situations, the stream’s locID may be represented in longitude and latitude coordinates using a GPS system. STDF discretizes these location coordinates, as discussed in the IoT-based Spatial Data Handler. For instance, to cluster a stream (data unit) which has the location coordinates (29.2392, 32.5983), STDF discretizes these location coordinates by classifying the 10 services’ location IDs in D1 into 10 intervals with the following cut points: ${\mathrm{LocID}}_{1}$: (29.1118, 32.6598); ${\mathrm{LocID}}_{2}$: (27.2579, 33.8116); ${\mathrm{LocID}}_{3}$: (25.0676, 34.8790); ${\mathrm{LocID}}_{4}$: (26.7500, 33.9360); ${\mathrm{LocID}}_{5}$: (27.9654, 34.3618); ${\mathrm{LocID}}_{6}$: (29.6725, 32.3370); ${\mathrm{LocID}}_{7}$: (29.5933, 32.7178); ${\mathrm{LocID}}_{8}$: (27.7833, 33.5666); ${\mathrm{LocID}}_{9}$: (27.0370, 33.8523); and ${\mathrm{LocID}}_{10}$: (26.8482, 33.9900). Therefore, by calculating the Euclidean-based distance between (29.2392, 32.5983) and the cut points, the distances are 0.141467, 2.323284, 4.754349, 2.825873, 2.175431, 0.505991, 0.373721, 1.748499, 2.534206 and 2.766534 for loc ID= 1, 2, 3, 4, 5, 6, 7, 8, 9 and 10, respectively. Thus, STDF discretizes the coordinates to ${\mathrm{LocID}}_{1}$ as per the minimum distance.

#### 5.4.2. Evaluating the Aggregation Scalability of STDF IoT Data Fusion

**Figure 12:**
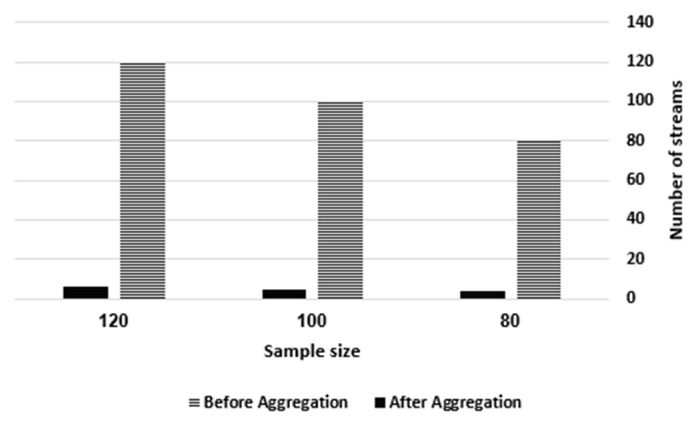
The number of aggregated streams vs. the sample size.

To manage scalable IoT data, the TAG technique aggregates the streams of a specific SourceID per its location IDs. Thus, this experiment tracked the number of streams with respect to the location IDs per SourceID before and after aggregation at the IoT-based Temporal Data Aggregator. Hence, we investigated the number of streams of the TAG tree of ${service}_{1}$ before and after aggregation at the sample sizes of 80, 100 and 120 over D1. There is no need to perform the same experiment over D2 or D3, as it is a factor of the sample size only. Table 5 presents the number of streams per Loc ID before aggregation. For instance, there are 80 streams at a sample size of 80, which are distributed through four location IDs as follows: 26, 21, 18 and 15. Using 100 streams at a sample size of 100, the streams are distributed over five location IDs as follows: 27, 22, 21, 17 and 13. At a sample size of 120, the streams are distributed over six location IDs as follows: 29, 23, 22, 17, 15 and 14. However, the total number of streams upon aggregating the freshest stream per location at a sample size of 80 is four streams (one stream per location ID); at a sample size of 100, the total number of streams is five streams; and at a sample size of 120, the total number of streams is six streams. Figure 12 shows the number of streams before and after aggregation, which emphasizes that the aggregation reduces the total number of streams per SourceID up to 95%.

#### 5.4.3. Evaluating the Spatiotemporality of STDF IoT Data Fusion

This experiment tracked the performance of both the IoT-based Spatial Data Handler and the IoT-based Temporal Data Aggregator on a time basis to examine the spatiotemporal IoT data processing time. We investigated their performance for ${service}_{1}$ over the first three seconds of the simulation. The streams are variable in their processed size, and they are randomly selected by the PPS sampling step. Thus, the same SourceID has a different number of location IDs every second, while the sample size is constant. As shown in Table 6, after sampling the streams of ${service}_{1}$ using a sample size of 80, the streams were transmitted from four, three and four location IDs at the first, second and third seconds, respectively. At a sample size of 100, the streams were transmitted from five location IDs over the three seconds, while at a sample size of 120, the streams were transmitted from six, five and six location IDs at the first, second and third seconds, respectively. Furthermore, each location ID transmitted a different number of streams every second, as the streams have different processed sizes despite the constant SourceID and sample size. After aggregating the streams of ${service}_{1}$ using a sample size of 80, four streams were transmitted from four location IDs at the first second, three streams were transmitted at the 2nd second and four streams were transmitted at the third second. At a sample size of 100, five streams were transmitted from five location IDs over the three seconds, while at a sample size of 120, six streams were transmitted at the first second, five streams were transmitted at the 2nd second and six streams were transmitted at the third second. This experiment proves that STDF temporally updates the spatial streams of one source ID.

#### 5.4.4. Evaluating the Real-Time Aggregation Processing of STDF IoT Data Fusion

**Figure 13:**
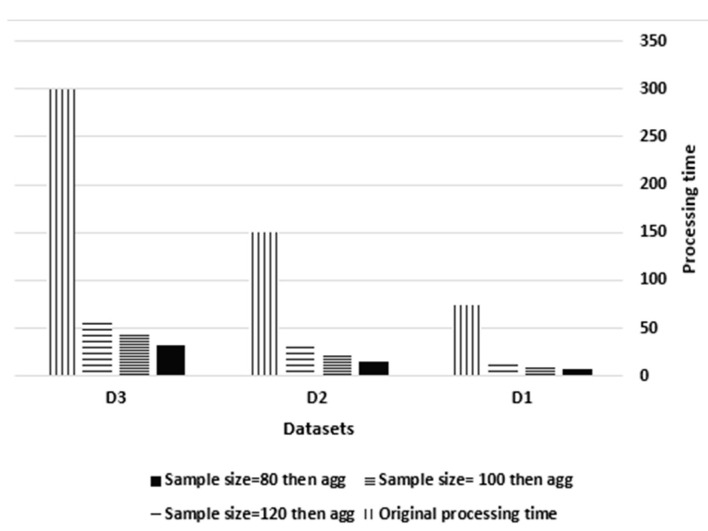
Processing time after aggregation vs. sample size.

The processing time of the simulator in the cloud data center was investigated before and after the aggregation of all source IDs through the three datasets per second to ensure the real-time IoT data processing of STDF. As shown in Figure 13, the original processing time of D1 with neither sampling nor aggregation was 75 s, while it became 7, 10 and 15 s when using sample sizes of 80, 100 and 120, respectively. Further aggregation reduced the original processing time by an average of 90%, 86% and 80%, respectively.

As for D2, the original processing time was 150 s, while it became 15, 21 and 32 s when using sample sizes of 80, 100 and 120, respectively. Further aggregation reduced the original processing time by an average of 90%, 85% and 81%, respectively. Regarding D3, the original processing time was 300 s, while it became 33, 45 and 60 s when using sample sizes of 80, 100 and 120, respectively. Further aggregation decreased the original processing time by an average of 90%, 85% and 81%, respectively.

### 5.5. STDF Performance Evaluation Compared to the Main IoT Data Fusion Approaches

This section presents a detailed comparison between the proposed STDF approach using the largest dataset (D3) and the main IoT data fusion approaches presented in Section 2. Table 7 provides a summarized description of the datasets used in those related works and thus considered in this section for the comparative evaluation. The comparison evaluates STDF’s performance with respect to the following aspects: (1) processing time, (2) accuracy level and (3) the considered IoT data perspectives. Each dataset is identified by its IoT domain that clarifies the IoT application, data size in gigabytes (GB), time span in seconds (s), features that indicate the nature of the dataset attributes, the modality, the specific considered IoT data dimensions that are involved in the dataset and the evaluation metric applied to the dataset, being either the processing time (PT) or accuracy level (AL). 

#### 5.5.1. Processing Time Evaluation

This section considers the maximum achieved processing time as the comparison metric between STDF and the related works that evaluated their performance using the processing time, as presented in Table 1. This includes comparing their dataset size, dataset nature and hardware specifications. The approach in [12] consumed 144 s using an 18-gigabyte biomedical dataset on a 2.00 GHz Intel Core i7-4510U CPU and 16 GB RAM. In [17], the approach consumed 86 s using an 8-gigabyte biomedical dataset on a machine with a 2.52 GHz CPU and 3.75 GB RAM. As for STDF, it consumed 60 s using our 12-gigabyte dataset (D3) on a Core i7, 2.70 GHz and 8 GB RAM.

#### 5.5.2. Accuracy Evaluation

**Figure 14:**
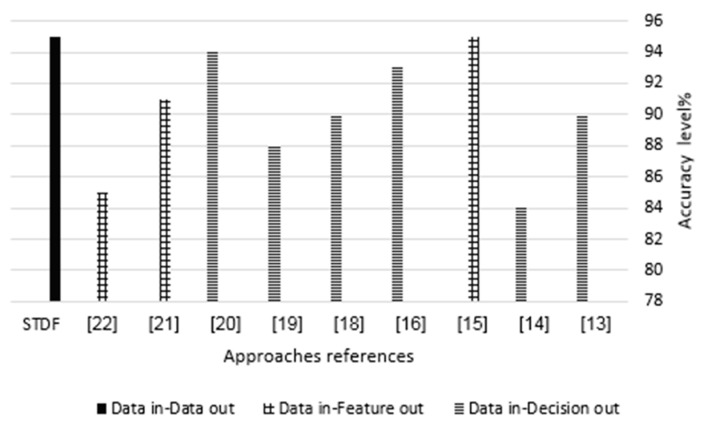
STDF accuracy evaluation.

This section compares the accuracy of STDF and the related works that considered the accuracy metric as per Table 1 and shown in Figure 14. The accuracy metric for the data fusion approaches depends on the used data fusion method/technique, irrespective of the IoT domain. 

Since STDF is a DAI–DAO fusion approach, we evaluated the accuracy of STDF using the formulas presented in (12), (13) and (14) that measure the data out accuracy that results from the double-stage sampling method. As clarified earlier in Section 2, the scope of our related works is low-level data fusion that focuses on low-level (raw) IoT data despite the fusion outcome being data, features or decisions. Therefore, the accuracy of the approaches in [15,21,22] would be the feature extraction accuracy, as they represent data in–feature out fusion approaches. The accuracy of the approaches in [13,14,16,18,19,20] is the decision making accuracy, as they represent data in–decision out fusion approaches. Figure 14 presents the maximum accuracy level achieved by each approach using the data fusion method mentioned in Table 1 as per the intended outcome for the fusion. An accuracy level of 90% was achieved for detection, 84% for energy pricing estimation, 95% for recognition, 93% for optimization, 90% for weather forecasting, 88% for detection, 94% for opinion clustering, 91% for recognition and 85% for feature extraction with respect to the approaches presented in [13,14,15,16,17,18,19,20,21,22], respectively. As for STDF, it achieved an accuracy level of 95% for the data out that resulted from the double-stage sampling method.

#### 5.5.3. Evaluation of IoT Data Perspectives

**Figure 15:**
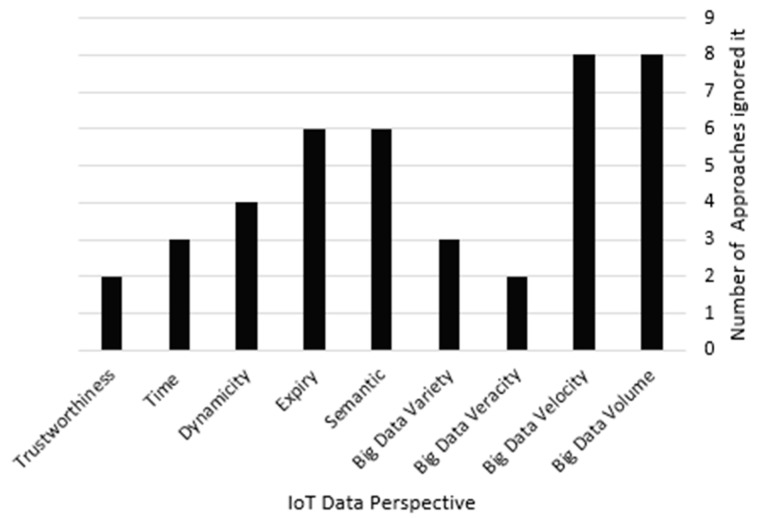
Evaluation of IoT data perspectives.

STDF and the related works performed low-level data fusion, which focuses on fusing raw IoT data streams. The main concern of this evaluation is to determine how many related studies overlooked the deduced challengeable IoT data perspectives discussed in Section 3, in order to decide their coverage capabilities and tolerance in the IoT environment. Thus, this section investigates how many times IoT data perspectives were ignored among the main related works’ approaches. Figure 15 presents every IoT data perspective and the number of related works’ approaches that ignored it. For example, the big data volume and velocity were ignored by eight approaches, whereas the big data veracity, big data variety, IoT data semantics, IoT data expiry, IoT data dynamicity, IoT data time and IoT data trustworthiness were ignored by two, three, six, six, four, three and two approaches, respectively, as detailed in Table 1. On the other hand, STDF considered seven IoT data perspectives, concerning the most neglected ones, such as the big data volume and big data velocity, while it bypassed only two perspectives, namely, the big data veracity and big data variety, since STDF does not consider the data nature as it is a DAI–DAO data fusion approach.

## 6. Discussion

Our experimental study conveys that STDF efficiently manages different IoT data processing challenges, such as security, reliability, scalability, accuracy and latency, through uniquely considering most of the IoT data perspectives such as the big data volume, big data velocity, IoT data semantics, IoT data expiry, IoT data dynamicity, IoT data time and IoT data trustworthiness, which are ignored by most of the previous IoT data fusion approaches, as shown in Figure 15. STDF ensures secure processing by conducting a validation review of the source IDs of all acquired streams before processing. It also ensures reliable processing by guaranteeing streams’ freshness over different time intervals. Since data freshness is domain dependent, the experimental evaluation of data freshness shows that increasing the interval of the freshness time increases the number of streams for processing. STDF preserves the spatial IoT data feature by clustering the streams of one service on their transmitting services to ensure reliable processing, assuming that any fault that occurs in any stream at a specific loc ID will affect only the streams at this loc ID rather than those at other locations. Considering the perspectives of the scalability and latency challenges, STDF utilizes both cluster sampling and aggregation techniques for data reduction. Table 8 summarizes the number of streams and processing times that resulted from experimenting with the combination of both techniques on dataset D3.

The original dataset D3 had 6424 streams and was processed in 300 s before reduction. Using cluster sampling, 3200, 4000 and 4800 streams were generated per second, with processing times of 100, 140 and 200 s with sample sizes of 80, 100 and 120, respectively. The summary table demonstrates that cluster sampling reduces the number of streams in the original dataset by an average of 50%, 40% and 35%, which decreases the original processing time by 70%, 55% and 34%, respectively. Furthermore, after utilizing aggregation for fusion, the number of streams was reduced to 167, 215 and 258, with processing times of 33, 45 and 60 s and sample sizes of 80, 100 and 120, respectively. Hence, aggregation reduces the sampled number of streams by an average of 95% and reduces the processing time by an average of 70% with respect to cluster sampling. 

Thus, applying sampling followed by aggregation using STDF returns the lowest processing time value compared to the main IoT data fusion approaches discussed in the Related Works section. Moreover, to ensure reliable processing, STDF updates the fusion scenario every second to grant the renewed fresh streams at the updated location IDs. Finally, accuracy is guaranteed in STDF via the cluster sampling accuracy that reaches its minimum value with an average of 85% at D1, which increases by increasing the dataset size, achieving the highest accuracy value compared to the main IoT data fusion approaches presented in the Related Works section. In addition, preserving the spatial IoT data feature by utilizing constant centroids while clustering the streams improves STDF’s accuracy.

Considering the conducted experiments and the obtained results, STDF is a DAI–DAO fusion approach that incorporates real problems and challenges. It handles data loss and outliers that result from sensor faults via the IoT Data Quality and Freshness Handler, it considers different location formats through the IoT-based Spatial Data Handler and it supports the processing of massive data amounts via the IoT Data Reducer module, appended with a state estimator for contingencies. Moreover, it guarantees real-time processing of data streams.

## 7. Conclusions and Future Work

Data fusion in IoT-based systems encounters many processing challenges due to the unprecedent features associated with IoT data. In this paper, we proposed the IoT-based spatiotemporal data fusion (STDF) approach as a domain-independent data in–data out (DAI–DAO) data fusion approach that maintains different IoT data features prior to any business analytics by introducing two layers: (1) the IoT-based Data Features Manager, and (2) the IoT-based Data Fusion Manager. The first layer directly proceeds after data acquisition to ensure the data trustworthiness, quality and freshness, and to reduce the IoT data volume using the cluster sampling technique, managing IoT processing security, reliability, accuracy, latency and scalability challenges. The second layer lies in the processing server to preserve the spatial IoT data feature using the k-means clustering technique and to reduce the IoT data volume using the Tiny AGgregation (TAG) technique by aggregating the IoT sources’ data in the freshest data per location. STDF’s processing scenario is repeated on a time basis to cope with the temporal IoT data feature. The experimental results of the proposed STDF approach indicate an efficient performance, where it reduced the IoT data volume by an average of 95% and decreased the processing time by an average of 80%, with a 90% average accuracy level for the largest used dataset. Our future work is to consider the variable IoT data multi-modality feature by extending the DAI–DAO STDF approach to fuse IoT data features, being dedicated to a specific domain, as well as considering high-level and middle-level data fusion.

## Figures and Tables

**Table 1:** Evaluation of data fusion methods in IoT systems from the big data and IoT perspectives.

Ref	Data Fusion Target	Processing Technology	Data Fusion Method	Handled Big Data Characteristics	Ignored IoT Data Perspective	Evaluation Metrics
[12]	Complex event pattern detection for smart healthcare	Cloud processing	Complex event processing	Variety, veracity and velocity	Data dynamicity and data expiry	Processing time
[13]	Data source anomaly detection for smart mobility	Cloud processing	Model-based estimation	Variety and veracity	Data trustworthiness, data semantics and data time	Accuracy using data variance
[14]	Real-time energy pricing for smart energy	Distributed processing system	Distributed state estimation	Velocity, veracity and variety	Data expiry	Accuracy using F-measure
[15]	Gesture recognition imagery	Central processing	Segmentation followed by classification	Volume and veracity	Data semantics and data expiry	Accuracy using recognition rate
[16]	Optimization of sensor data allocation	Central processing	Particle swarm optimization	Veracity and variety	Data semantics and data time	-
[17]	Body movement monitoring for smart healthcare	Central processing	CNNs	Veracity and volume	Data semantics and data expiry	Processing time
[18]	Weather forecasting for smart environments	Cloud processing	Kriging with external drift	Variety	Data semantics	Accuracy using mean error
[19]	Nuclear power crack detection	Central processing	CNNs and NB	-	Data semantics	Accuracy using root mean square error
[20]	Reputation generation and opinion mining	Central processing	Data clustering	Variety and veracity	Data expiry and data time	Accuracy using data deviation
[21]	Automatic Modulation recognition	Central processing	Global average and max pooling	Variety and veracity	Data expiry and data trustworthiness	Classification accuracy
[22]	Video summarization	Cloud processing	CNNs	Volume, variety, velocity and veracity	Data semantics	Accuracy using F-measure

**Table 2:** Tracking the D1 services’ trust degrees.

Service Number j	$PR_{1j}$	$WT_{1}$	$PR_{2j}$	$WT_{2}$	$PR_{3j}$	$WT_{3}$	$T_{j}=\overset{i=3}{\underset{i=1}{\sum }}WT_{i}\cdot PR_{ij}$
1	8	0.2	2000	0.4	2500	0.4	1801.6
2	3	0.2	800	0.4	1000	0.4	720.6
3	5	0.2	750	0.4	600	0.4	541
4	4	0.2	1000	0.4	1300	0.4	920.8
5	2.5	0.2	600	0.4	850	0.4	580.5
6	0.5	0.2	1100	0.4	1400	0.4	1000.1
7	1.5	0.2	350	0.4	650	0.4	400.3
8	3	0.2	650	0.4	900	0.4	620.6
9	2	0.2	850	0.4	1050	0.4	760.4
10	4.5	0.2	1150	0.4	1350	0.4	1000.9

**Table 3:** Tracking PPS parameters per second at a sample size of 80 for D1.

Service Number	a	b	d	c	$P_{1}=(a*d)/b\;\;$	$P_{2}=c/a\;$	$W=1/\left(P_{1}*P_{2}\right)\;$	Processed Size Mean
1	139	1606	10	80	0.865504359	0.575539568	2.0075	327
2	167	1606	10	80	1.03985056	0.479041916	2.0075	287
3	161	1606	10	80	1.00249066	0.49689441	2.0075	291
4	149	1606	10	80	0.927770859	0.536912752	2.0075	312
5	167	1606	10	80	1.03985056	0.479041916	2.0075	278
6	174	1606	10	80	1.083437111	0.459770115	2.0075	264
7	167	1606	10	80	1.03985056	0.479041916	2.0075	274
8	154	1606	10	80	0.95890411	0.519480519	2.0075	298
9	164	1606	10	80	1.02117061	0.487804878	2.0075	277
10	164	1606	10	80	1.02117061	0.487804878	2.0075	280

**Table 4:** The location IDs of service_1_’s streams at sample sizes of 80, 100 and 120 for dataset D1.

Dataset	Sample Size	Number of Location IDs	Location IDs
D1	80	4	$LocID_{1}$ $LocID_{2}$ $\;LocID_{3}$ $LocID_{4}$
	100	5	$LocID_{1}$ $LocID_{2}$ $\;LocID_{3}$ $LocID_{4}$ $LocID_{5}$
	120	6	$LocID_{1}$ $LocID_{2}$ $\;LocID_{3}$ $LocID_{4}$ $LocID_{5}$ $LocID_{6}$

**Table 5:** The distribution of the streams of service_1_ as per the location IDs at sample sizes of 80, 100 and 120 before aggregation for dataset D1.

Dataset	Sample Size (Number of Streams per Source ID)	Number of Location IDs per Source ID	Streams per Location ID					
			$LocID_{1}$	$LocID_{2}$	$\;LocID_{3}$	$LocID_{4}$	$LocID_{5}$	$LocID_{6}$
D1	80	4	26	21	18	15	-	-
	100	5	27	22	21	17	13	-
	120	6	29	23	22	17	15	14

**Table 6:** Three seconds of service1’s simulation regarding the IoT-based Spatial Data Handler and the IoT-based Temporal Data Aggregator.

Second	Sample Size (# of Streams per Source ID before Aggregation)	Number of Location IDs per Source ID	Streams per Location ID						# Streams per Source ID after Aggregation
			$LocID_{1}$	$LocID_{2}$	$LocID_{3}$	$LocID_{4}$	$LocID_{5}$	$LocID_{6}$	
s1	80	4	26	21	18	15	-	-	4
	100	5	27	22	21	17	13	-	5
	120	6	29	23	22	17	15	14	6
s2	80	3	32	25	23	-	-	-	3
	100	5	31	27	23	15	4	-	5
	120	5	32	29	21	23	15	-	5
s3	80	4	25	19	23	13	-	-	4
	100	5	25	25	20	15	15	-	5
	120\	6	27	23	24	14	20	12	6

**Table 7:** Summarized description of the main considered datasets for the comparative evaluation.

Ref#	IoT Domain	Size in GB	Time Span in s	Dataset Features	Dataset Modality	Considered IoT Data Dimensions	Evaluation Metric (AL or PT)
[12]	Smart healthcare	18	60	Sensor and remote server specifications	Structured	Fast generated, imprecise, diverse, private, informative and temporal data	PT = 144 s
[13]	Smart mobility	12	-	Sensor specifications	Structured	Imprecise, diverse, informative and spatial data	AL = 90%
[14]	Smart energy	16	60	Sensor specifications	Structured	Fast generated, imprecise, diverse, temporal and spatial data	AL = 84%
[15]	Domain independent	8	10	Image and pixel specifications	Images	Massive, diverse, private, temporal and spatial data	AL = 95%
[16]	Smart home	9	-	Sensor specifications	Structured	Imprecise, diverse, private and spatial data	AL = 93%
[17]	Smart healthcare	8	60	Image specifications	Images	Massive, diverse, private, temporal and spatial data	PT = 86 s
[18]	Smart environments	13.5	60	Sensor specifications	Structured	Imprecise, volatile, private, temporal and spatial data	AL = 90%
[19]	Smart energy	14	60	Sensor specifications	Structured	Volatile, private, temporal and spatial data	AL = 88%
[20]	Smart social networks	7	-	Network specifications	Structured	Imprecise, diverse, spatial, informative and private data	AL = 94%
[21]	Smart wireless communication	12	60	Sensor specifications	Structured	Imprecise, diverse, temporal, spatial and informative data	AL = 91%
[22]	Domain independent	22	10	Video specifications	Videos	Imprecise, massive, fast generated, diverse, temporal, volatile, private and spatial data	AL = 85%
STDF	DAI–DAO domain independent (D3)	12	15	Stream specifications	Structured	Fast generated, massive, volatile, private, informative, temporal and spatial data	AL = 95% and PT = 60 s

**Table 8:** Comparison of data reduction techniques for dataset D3.

Reduction Technique	Sample Size	Number of Streams	Processing Time (Seconds)
Without reduction	-	6424	300
Cluster sampling before aggregation	80	3200	100
	100	4000	140
	120	4800	200
Cluster sampling followed by aggregation	80	167	33
	100	215	45
	120	258	60

## Data Availability

Not applicable.
